# Case Report: Transoral Penetrating Medullocervical Injury by a Chopstick in Three Cats

**DOI:** 10.3389/fvets.2020.609869

**Published:** 2020-12-16

**Authors:** Yukiko Nakano, Yuta Nozue, Kohei Nakata, Toru Fujioka, Yoichi Sakai, Hiroaki Kamishina

**Affiliations:** ^1^The Animal Medical Center of Gifu University, Gifu University, Gifu, Japan; ^2^The United Graduate School of Veterinary Sciences, Gifu University, Gifu, Japan; ^3^Ivy Animal Clinic, Okayama, Japan; ^4^Nishikani Animal Hospital, Gifu, Japan; ^5^Center for Highly Advanced Integration of Nano and Life Sciences, Gifu University, Gifu, Japan

**Keywords:** penetrating injury, chopstick, atlantooccipital joint, CT, MRI, surgical management

## Abstract

This case series describes transoral penetrating or contusive medullocervical injury by a wooden chopstick in three young cats presenting with acute tetraparesis. CT revealed that remnant fragments of a wooden chopstick penetrated the atlantooccipital space in cases 1 and 2. The remnant fragments were visualized clearly on CT under the bone window setting. MRI revealed a hyper-intense lesion in the spinal cord parenchyma at the level of C1 on T2-weighted images in case 3. Tetraparesis improved after surgical removal of the remnant fragment in case 2 and with supportive care in case 3.

## Introduction

In humans, penetrating injuries by chopsticks have been sporadically reported in Asian nations because of their cultural background (1–5). Although craniocerebral penetrating injures by chopsticks have been reported in both adults and pediatric patients, most accidents occur in children and are caused by accidental fall (2, 3). Chopsticks can penetrate from various entry sites, such as transobital, transoral, transnasal, and transbuccal, and *via* the temporal bone. The most common entry site is the orbit, followed by the oral cavity (3). Injuries by wooden chopsticks often result in more severe brain damage than metal chopsticks because the diameter of wooden chopsticks is larger (3). Wooden chopsticks may also break, thus the remnant fragment can cause infection and abscess (2).

In veterinary medicine, thoracic and abdominal penetrating injuries in cats were previously reported (6), but there is no report of penetrating injuries to the spinal cord by chopsticks. This is the first case series of penetrating injuries of the medullocervical region by chopsticks, which describes the clinical abnormalities, diagnostic findings, treatment, and outcome in three young cats.

## Case Description

### Case 1

**Figure 1:**
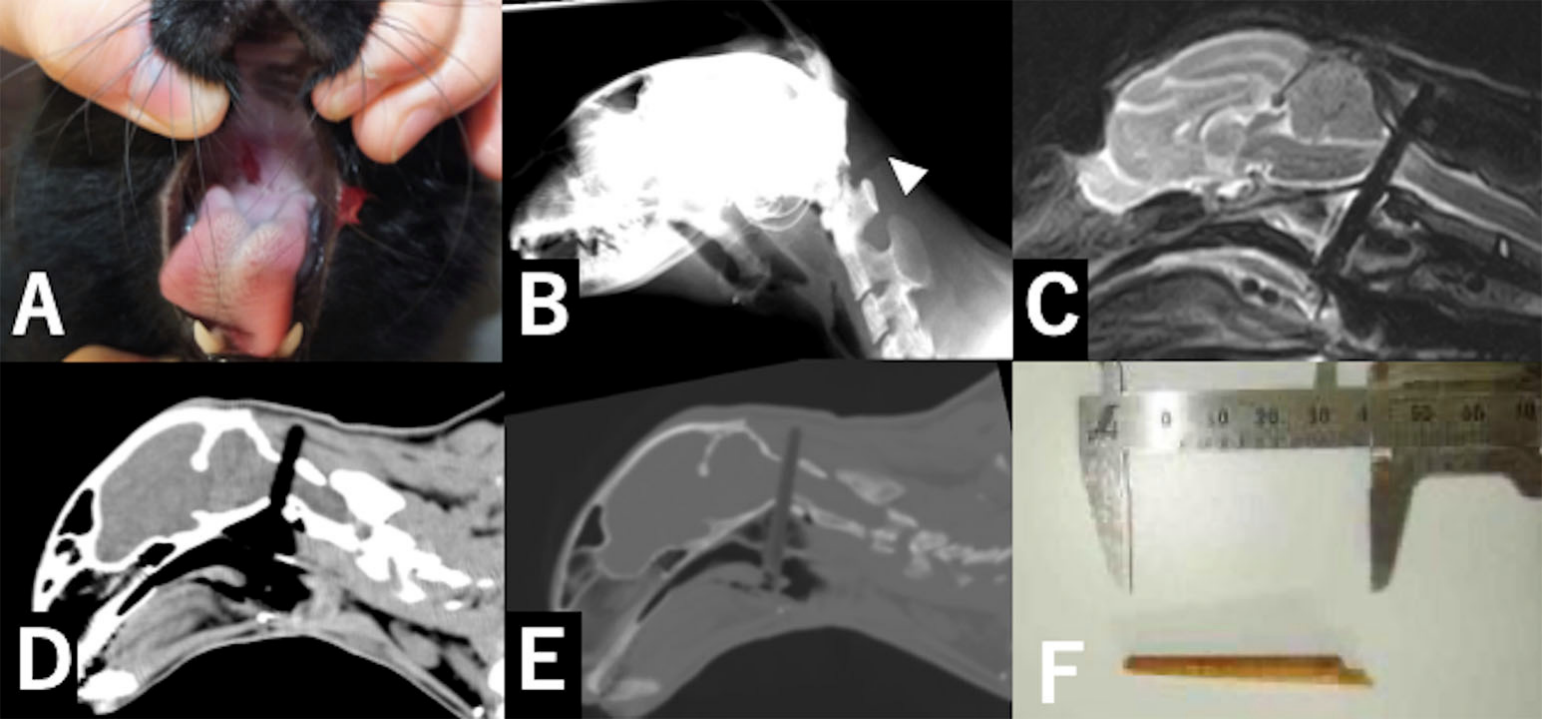
Pictures and diagnostic images in case 1. A small hole was found in the soft palate in the oral cavity. **(A)**. Lateral craniocervical radiography in case 1 **(B)**. A linear radiolucent area (arrowhead) was found in soft tissues dorsal to the atlantooccipital joint. A sagittal section of the craniocervical T2-weighted image **(C)**. A hypodense linear object penetrated the spinal cord. The sagittal section on craniocervical CT imaging with the soft-tissue setting (WL: 40, WW: 350) **(D)** and bone window setting (WL: 50, WW: 2000) **(E)**. A linear low-attenuation area penetrated the vertebral canal. The chopstick fragment was more clearly differentiated from air with the bone window setting than with the soft-tissue window setting **(C,D)**. The fragment of the chopstick was removed from the oral cavity. The fragment was consistent with the lost part of the broken chopstick **(F)**.

A 5-months-old neutered male domestic short hair was evaluated at Ivy Animal Clinic because of acute tetraparesis. According to the owners, the cat lied down on the floor beside a broken chopstick on the day of injury. The owner also claimed that the chopstick was shorter and the tip of the chopstick was missing. On presentation, the animal had difficulty raising his head and was unable to stand. On physical examination, a small hole was found at the soft palate in the oral cavity (Figure 1A). Neurological examination revealed non-ambulatory tetraparesis and that the proprioception of four limbs were absent and the spinal reflexes were increased. The mental status and results of cranial nerve examination were normal. Based on the neurological signs, the location of the lesion was suspected to be the C1–C5 spinal cord segment. The clinical history suggested the differential diagnosis included trauma, congenital disease and vascular disease. Radiography of the craniocervical region revealed a radiolucent area in the dorsal soft tissue of the atlantooccipital joint (Figure 1B).

Computed tomography (CT) and magnetic resonance imaging (MRI) were performed without sedation or general anesthesia using a multi-detector CT scanner (ECLOS-8S, Hitachi Healthcare, Tokyo, Japan) and 0.3-T MR scanner (AIRIS Vento LT, Hitachi Healthcare, Tokyo, Japan). The cat was positioned lateral recumbency on a vacuum beanbag when MRI examination was performed. CT revealed a hypodense linear structure penetrating the vertebral canal from the oral cavity passing through the atlantooccipital space. The Hounsfield Unit of the linear structure was ~−300 HU. The density of the structure was similar to that of air with the soft-tissue window setting (window level: 50 HU, window width: 350 HU); however, the structure was distinguished easier from air with the bone window setting (window level: 400 HU, window width: 4,000 HU) than with the soft-tissue window setting (Figures 1D,E). On MRI, the structure was visualized as a linear signal void area and the spinal cord surrounding the linear structure was hyper-intense on sagittal T2-weighted images (TE: 125 ms, TR:3,900 ms) (Figure 1C). The imaging findings and clinical history suggested medullocervical penetrating injury by a chopstick, and that the tip of the chopstick remained inside the wound. Initial treatment included dexamethasone (0.2 mg/kg, Dexium, Bimeda USA, Ilinois), tranexiamic acid (10 mg/kg, Transamin injection, Daiichi-Sankyo, Tokyo), trimethoprim-sulphadiazine combination (trimethoprim 4 mg/kg, sulphadiazine 20 mg/kg, Tribrissen, Kyoritsu Seiyaku Corporation, Tokyo), and cefovesin sodium (8 mg/kg, Convenia, Zoetis Japan, Tokyo) administration subcutaneously, and intravenous infusion of a maintenance solution (3 ml/kg/hr, SOLDEM ®3A, TERUMO Corporation, Tokyo, Japan).

Surgical removal of the chopstick was planned and supportive treatment was continued, but respiration arrested suddenly and the cat died 2 days after the injury. After death, the tip of the chopstick (3.5 cm length) was found and removed from the hole of the soft palate (Figure 1F).

### Case 2

**Figure 2:**
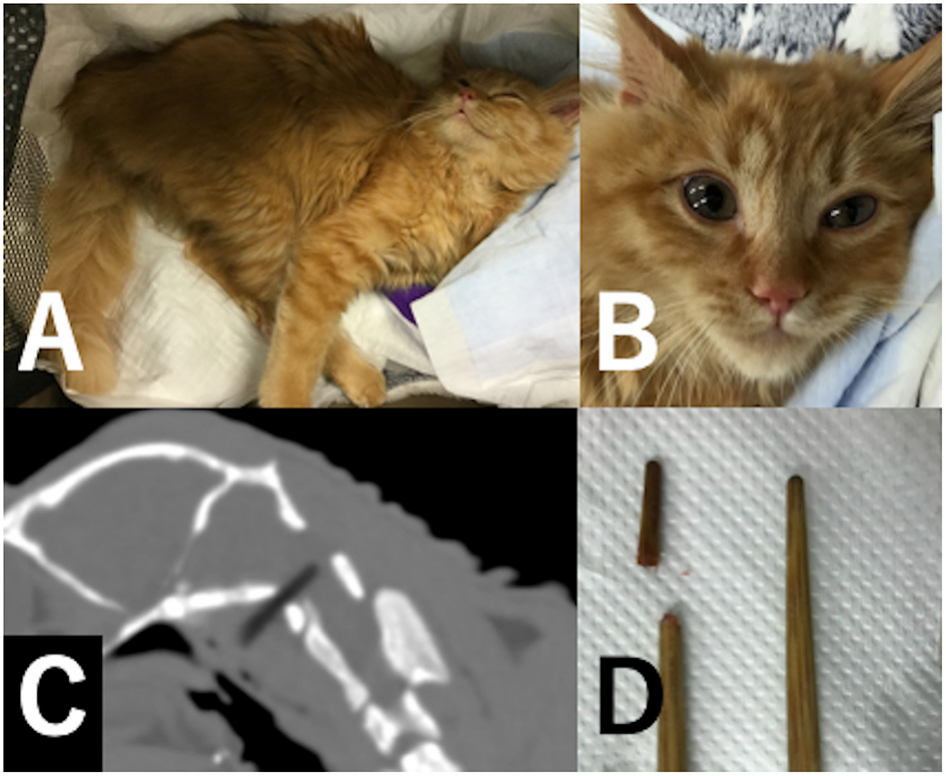
Pictures and diagnostic images in case 2. At presentation, a decreased level of consciousness, tetraparesis **(A)**, and Horner's syndrome **(B)** were observed. A low-attenuation linear structure from the oral cavity passing through the atlantooccipital space was found on CT examination **(C)**. The fragment of the chopstick was removed surgically **(D)**.

A 5-months-old intact male domestic long-haired cat was referred to the Animal Medical Center of Gifu University 10 h after the acute onset of tetraparesis. The owners heard something falling and found the cat struggling on the floor beside a chopstick. The chopstick was revealed to be shorter by the medical interview. The mental status of the cat was comatose since after onset of tetraparesis. Initial treatment included subcutaneous injection of prednisolone (1 mg/kg, prednisolone injectable solution “KS,” Kyoritsu Seiyaku Corporation, Tokyo, Japan) and intravenous injection of mannitol (1 g/kg, mannitol injection, Yoshindo, Toyama, Japan). At presentation, the mental status of the cat was obtundation and the cat was unable to stand (Figure 2A). Neurological examination revealed non-ambulatory tetraparesis, left miosis and ptosis, and protrusion of the third eyelid suggesting Horner's syndrome (Figure 2B). The proprioceptions of the four limbs were absent and the spinal reflexes were increased. The neurological signs localized the lesion to the left-sided brainstem and/or C1-5 spinal cord segment lesion. The clinical history suggested penetrating trauma by a chopstick and the tip of the chopstick was suspected to remain in the lesion.

CT examination was performed without sedation or general anesthesia using a multi-detector CT scanner (Alexion, Canon Medical Systems Corporation). CT revealed a low-attenuation linear structure from the oral cavity passing through the atlantooccipital space. The tip of the linear structure was inside the vertebral canal. The Hounsfield Unit of the linear structure was −350 HU. The wooden foreign body was distinguished from air easier with the bone window setting (Figure 2C).

Surgical removal was performed on the day following presentation. Anesthesia was induced with propofol at 10 mg/kg by IV (PropoFlo 28, Zoetis Japan, Tokyo) and maintained with sevoflurane (3.0 %, SevoFlo, Zoetis Japan, Tokyo) in oxygen. The perioperative analgesia consisted of fentanyl citrate by continuous infusion (CRI; 0.005–0.02 mg/kg/h, Fentanyl Injection, Janssen Pharmaceutical KK, Tokyo). The perioperative antibiotic therapy consisted of cefazolin (20 mg/kg by IV, Cefamezin α, LTL Pharma, Tokyo). The cat was positioned in dorsal recumbency and the neck was slightly extended. The forelimbs were retracted caudally. A cranio-cervical ventral midline incision was made. The sternohyoideus muscle was separated in the middle, and the trachea with the thyroid grand, right sternothyroideus muscle, and right common carotid artery sheath was exposed, and separated by blunt resection. The trachea and the right sternothyroideus muscle were retracted to the left side, and the right common carotid artery sheath was retracted to the right side using Gelpi retractors. The longus capitis muscles were exposed and the tip of the chopstick penetrating the atlantooccipital space was found. The chopstick was carefully pulled out using forceps. When the chopstick was pulled out, a small amount of purulent matter flowed out surrounding the chopstick. After the tip of the chopstick was removed, blood flowed away from the small hole in which the tip of the chopstick was stuck. Bleeding was stopped by plugging the hole with a small absorbable gelatin sponge (Spongel, LTL Pharma, Tokyo). The wound was thoroughly irrigated and closed in a routine manner. After surgery, the cat was administered fentanyl citrate CRI (0.003 mg/kg/h) as a postoperative analgesic for 24 h, and cefazolin (20 mg/kg by IV per 12 h) as a postoperative antibiotic until the cat was discharged. The fragment was consistent with the lost part of the chopstick (Figure 2D). The cat recovered uneventfully and was discharged 2 days after surgery. After discharge, oral administration of cefalexin (20 mg/kg per 12 h,) was continued for 1 month. At 2 weeks postoperatively, tetraparesis and Hornor's syndrome resolved.

### Case 3

**Figure 3:**
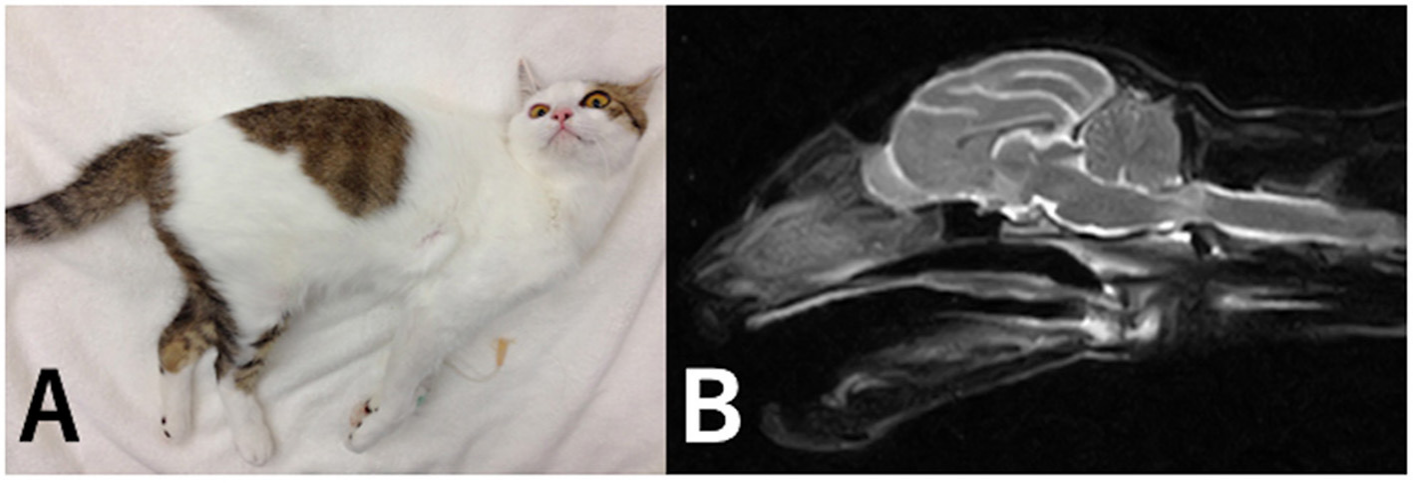
Picture and MR image in case 3. At the time of referral, the cat exhibited tetraparesis **(A)**. A hyper-intense lesion in the spinal cord parenchyma was identified at the level of C1 on T2-weighted sagittal images **(B)**.

A 15-months-old spayed female domestic short-haired cat was brought to Nishikani Animal Hospital. According to the owner, the cat became unable to stand after dropping from a height of ~1 meter with a chopstick in her mouth. The owner reported that the unbroken chopstick was found nearby the cat. The cat also presented coma, breathing difficulty, and cyanosis. The pupils were also dilated. The cat was immediately intubated and managed by artificial respiration, and respiration failure resolved the following day. The cat was treated using prednisolone, cefazolin, and mannitol, and referred to the Animal Medical Center of Gifu University 2 days after initial treatment at the primary care clinic. Evaluation at the time of referral revealed non-ambulatory tetraparesis and head turning to the right. The mental status was normal. The proprioceptions of the four limbs were reduced and the spinal reflexes of the four limbs were increased (Figure 3A). The neurological signs localized the lesion to the right-sided brainstem and/or C1-5 spinal cord segment lesion. The clinical history suggested penetrating or contusive injury by a chopstick. Radiography of the craniocervical region was unremarkable.

CT and MRI were performed under general anesthesia. Induction of the anesthesia was performed using propofol and anesthesia was maintained using isoflurane (IsoFlo Zoetis Japan, Tokyo). During intubation, a small hole was found in the soft palate. CT revealed no craniocervical lesion. MRI revealed a hyper-intense lesion in the spinal cord parenchyma at the level of C1 on T2-weighted images (Figure 3B). No chopstick fragments were found on imaging. The cat was treated using prednisolone at 1 mg/kg q24 and cefalexin at 20 mg/kg q12h. Cefalexin was administered for 7 days. Prednisolone was tapered and stopped 2 weeks after presentation. Tetraparesis gradually improved and the cat was ambulatory with mild ataxia of the four limbs at 1 month after the trauma.

## Discussion

Although penetrating head injuries associated with chopsticks are rare in humans, they have been sporadically reported in Asian nations (1–5). Most penetrating head injuries with chopsticks occurred in pediatric patients and the mean age of patients was 3.7 years (3). The most common causes of accidents of pediatric cases were accidental fall (2, 3). Transoral penetration injuries were comparatively rare, and one pediatric case of penetration from the soft palate and atlantooccipital space was reported (3, 4). In our cases, the accidents occurred by young cats and were suggested to be accidental fall. Chopsticks have the potential to harm immature cats that prefer to play with them. The entry sites of chopsticks in our cases were the oral cavity and atlantooccipital space. Transoral penetration of foreign bodies through the atlantooccipital space may occur more frequently in young cats than in humans because humans hold chopsticks in their hands, whereas cats hold them with their mouth.

In order to visualize the chopstick fragments, it is important to choose appropriate imaging modalities. Wooden foreign bodies are rarely visualized on plain radiography and are often overlooked (7, 8). In our cases, although the chopstick that penetrated the soft tissue was observed as a linear hypodense area, that remained only in the vertebral canal was unable to be confirmed on plain radiography. For humans, CT is one of the best modalities to visualize wooden foreign bodies (7, 9). Dry wooden foreign bodies have a low Hounsfield Unit close to air in the acute state and may be confused with gas accumulation, which is a common post-traumatic finding under the soft-tissue window setting. To differentiate simple gas collection from wooden foreign bodies, the wide-bone window setting can be useful because the wood matrix can be revealed (9). The Hounsfield Units of the chopstick remnant fragment in cases 1 and 2 were ~−300 and −350 HU, respectively, higher than that of air (−900 HU) and lower than that of soft tissues such as muscles (~60 HU). In order to visualize the difference between the Hounsfield Unit of chopstick remnants and that of air, wide window width settings (~4,000) may be needed.

MRI is also a useful modality to visualize wooden foreign bodies (7, 9). Dry wooden foreign bodies are found as hypointense structures on T2- and T1-weighted images (7). MRI revealed the chopstick fragments as signal void area rather than hypointense linear structures in cases 1. In case 3, although no signal void area or hypointense structures were found, MRI revealed a hyper-intense lesion in the spinal parenchyma at the level of C1 on T2-weighted images. This suggested that no chopstick fragment remained, but that a chopstick entered the vertebral canal from the atlantooccipital space and damaged the spinal cord. MRI may also be useful to evaluate brain and spinal cord parenchyma edema and inflammation from penetrating or contusive injuries in patients without chopstick fragments. It may be also important to pay attention to the owner's description in order to find fragments. In cases 1 and 2 in which the fragments were found, the owner claimed that the chopsticks were shorter and the tips were missing. In contrast, the owner of case 3 reported that the chopstick was unbroken and no remnant fragments were found.

Penetrating injury with a wooden foreign body may cause vascular damage, wound infection, and cerebrospinal fluid leakage (2, 3). Wooden chopstick fragments may cause severe intracranial infection such as meningitis and intracranial abscess (2, 3). Large intracranial abscesses as a complication of penetrating injury with wooden chopsticks are a risk factor for subsequent complications such as mental deficits (10). For these reasons, wooden chopstick fragments must be searched carefully and removed completely as quickly as possible because severe neurological damage may remain if intracranial abscesses expand (2).

To surgically remove chopstick fragments, we approached via a cranio-cervical ventral midline incision. We were able to easily access the entry site of the chopstick fragment by this approach; therefore, it may be an adequate method to remove chopstick fragments entering through the atlantooccipital space. In cases 1 and 2, the chopstick fragments remained in the vertebral canal. In case 2, surgical removal of the fragment was carried out 2 days after the injury and the prognosis was favorable, whereas the cat in case 1 did not undergo surgery and died. The prognosis of penetrating injuries with chopstick fragments may be good if surgical treatment is performed quickly after injury. As in case 3 with no remaining chopstick fragment, the prognosis may be good by performing supportive treatment immediately after injury.

## Data Availability Statement

The original contributions presented in the study are included in the article/supplementary materials, further inquiries can be directed to the corresponding author/s.

## Author Contributions

YNa, YNo, KN, TF, YS, and HK helped with the diagnosis and participated in clinical case management. YNa and HK participated in the review and editing of the manuscript. All authors contributed to the article and approved the submitted version.

## Conflict of Interest

The authors declare that the research was conducted in the absence of any commercial or financial relationships that could be construed as a potential conflict of interest.
